# Modeling music student teachers’ behavioral intention of using artificial intelligence in China

**DOI:** 10.3389/fpsyg.2026.1756135

**Published:** 2026-01-29

**Authors:** Yanlong Niu

**Affiliations:** School of Music and Dance, Zhengzhou University of Science and Technology, Zhengzhou, China

**Keywords:** AI use, behavioral intention, pre-service music teachers, teacher education, the extended UTAUT model

## Abstract

**Introduction:**

The integration of artificial intelligence (AI) into education is rapidly increasing worldwide and governments actively promote teachers’ positive attitudes toward AI and its use in instructional practices. Although prior research has highlighted the potential of AI in music education, limited studies have examined the factors influencing pre-service music teachers’ intentions to use AI in teaching.

**Methods:**

This study employed an online questionnaire based on an extended Unified Theory of Acceptance and Use of Technology (UTAUT) model. A total of 370 pre-service music teachers participated in the survey, and structural equation modeling was used to examine the determinants of their intentions to integrate AI into teaching.

**Results:**

The proposed UTAUT model explained 62.4% of the variance in pre-service music teachers’ intentions to use AI. The results indicated that social influence, performance expectancy, and effort expectancy positively predicted intentions to use AI, whereas education policy and facilitating conditions had negative direct effects. AI usage habit showed no significant effect. Notably, education policy demonstrated positive indirect effects through effort expectancy and social influence, indicating a dual mechanism of policy influence.

**Discussion:**

The findings of this study provide insights into how individual, institutional, and policy-related factors jointly shape pre-service music teachers’ intentions to adopt AI in education. This study then discussed implications for AI in music teacher training programs.

## Introduction

Recently, artificial intelligence (AI) becomes increasingly important in everyday life and it has influenced many facets including education (Ramnarain et al., 2024). AI technologies (e.g., dynamic assessments, machine learning) have been widely introduced into education and it reshapes teachers’ practices (Zhang and Aslan, 2021). Furthermore, the emergence of generative AI intensifies educational practitioners’ interests as it can be recognized as an effective tool for teaching and reduce the burdens of both students and teachers (Zhai et al., 2021). For instance, AI-powered chatbots can provide learners with personalized learning experiences (Zhang et al., 2023) and automate administrative tasks for teachers (Li and Wang, 2024). Except for the significant opportunities that AI presents, researchers have also voiced some concerns about AI use in education. For teachers, Edwards and Cheok (2018) proposed that robots with AI might play some roles of teachers so that teacher shortage can be relieved, while Lacity and Willcocks (2017) cautioned that AI may challenge or replace teachers’ positions. For students, despite being considered as digital citizens, they may develop negative learning attitudes if they struggle to apply AI tools effectively within specific learning contexts (Ijaz et al., 2017). Moreover, there are some popular assumptions that AI will transform education but some researchers found it not always operates as planned and the evidence of its transformative influences remains limited (Holmes et al., 2022; Williamson, 2024). Ethics and privacy concerns also trigger ongoing discussions on AI integration in education among educators and researchers (Hagendorff, 2020; Abrams et al., 2019). Such controversies may develop teachers’ resistance and uncertainties toward using AI. Therefore, it is important for future studies to examine and explore teachers’ perceptions of AI integration into education.

AI in music education has received growing attention recently. Music education is a subject that includes both artistic and technical elements (Han, 2025) and music technology encompasses a combination of artistic creativity and technological innovation (Kladder, 2020). Existing studies suggest that AI integration can enhance music teachers’ teaching efficiency, alleviate music teacher shortages, reduce their workload, support individualized assessment and facilitate music teacher training (Huang and Ding, 2022; Cheng and Qu, 2025; Xi, 2023). AI technologies can provide personalized recommendations and adjustments to meet diverse music learners’ needs (Gao and Liu, 2023). For example, Xu and Chen (2023) developed AI-based personalized recommendation systems to improve music students’ learning efficiency. Li and Wang (2024) found that AI-powered chatbots into piano classes result in an increase of students’ academic performance. Han (2025) claimed that relying heavily on teachers’ subjective judgments is one of limitations of traditional music assessment systems. Previous studies found that introducing AI-driven evaluation tools can offer more accurate and objective feedback on students’ performance quality (Hosseini et al., 2024; Xuan, 2022; Ahmadian Yazdi et al., 2022). Additionally, to enhance the online security of music teaching, Zhang et al. (2024) introduced a deep neural network system for intrusion detection. Collectively, these findings demonstrate the considerable benefits of AI to improve music education and enrich students’ learning outcomes. However, more attention were emphasized on its implementation among in-service teachers, little has been done on exploring student teachers’ AI integration into education and AI in teacher education (Zhang et al., 2023).

Preparing student teachers to effectively apply AI technologies has become a pressing issue in educational research and practice. Tondeur et al. (2017) pointed out that unlike in-service teachers, student teachers were still developing their professional identities, pedagogical beliefs and educational technological competencies. It is challenging to equip student teachers for AI-enhanced teaching (Pedró et al., 2019). The successful AI use in teaching depends on student teachers’ perceptions and behavioral intention toward it (Su and Yang, 2022). Exploring the determinants of their willingness of applying AI is therefore crucial to make use of advantages of AI in education (Khlaif, 2018). Given the potential value of implementing AI in music teaching and the limited research on music student teachers’ AI use. This study was based on the UTAUT model to explore the perceptions and behavioral intentions of music student teachers toward AI integration. The research questions that this study addresses are listed below:

1. What are the determinants that affect music student teachers’ willingness toward AI in education?
2. What is the mechanism of music student teachers’ behavioral intentions of AI use?

### Technology integration in music teacher education in China

Chinese music student teachers typically receive their training through music departments within universities or conservatories of music (Mei and Yang, 2021). With the rapid advancement of digitalization nationwide, music education with technology assistance has been increasingly emphasized in national educational reforms. Chinese policymakers hope the integration of technologies will elevate music education across the whole country (Ministry of Education, 2018). According to the Ministry of Education’s guidelines (2011), the curriculum for music teacher education should not only strengthen student teachers’ musical knowledge and skills but also enhance their professional competencies such as digital skills. However, as there is no official national guidelines of the content of technology training, technology training programs for music teachers in China exist considerable differences across educational institutions (Guan and Ren, 2021). Moreover, research on intelligent classrooms in China began relatively late and studies focusing on AI use in music teaching remain underdeveloped. Existing literature tends to put more emphasis on evaluating the effectiveness of particular AI tools in music classrooms rather than the process of AI use in music teacher education programs (Guo et al., 2023; Li and Wang, 2024; Yan and Xia, 2024). He and Ren (2025) further highlight that Chinese music student teachers should learn to utilize emerging technologies to support student-centred teaching practices. This underscores the urgent requirement for more investigation of music student teachers’ usage of AI.

### Proposed research model and research hypotheses

To investigate music student teachers’ intentions of AI implementation in teaching, this study grounded in UTAUT model, which is widely applied to explore individuals’ reactions to and application of emerging technologies (Venkatesh et al., 2012; Lameras and Arnab, 2021). It combines constructs from eight foundational models such as the theory of planned behavior (TPB), the technology acceptance model (TAM) and the innovation diffusion theory (IDT) (Venkatesh et al., 2003). The original UTAUT model comprises four core determinants of individuals’ willingness of using technology: namely performance expectancy, effort expectancy, social influence and facilitating conditions (Zhou et al., 2022). In recent years, numerous empirical studies have indicated the model’s effectiveness in explaining and predicting individuals’ intentions to use emerging technologies in educational contexts (An et al., 2023; Kittinger and Law, 2024). Therefore, UTAUT model is particularly suitable for understanding teachers’ acceptance of novel technologies such as AI.

However, there are still limited research of applying UTAUT within music education, especially in non-Western contexts (Zhang et al., 2021). Technology adoption is not only an individual behavioral process but also embedded within boarder institutional and policy environments. In collectivistic cultural contexts and policy-driven systems such as China, education policy plays a crucial role in the improvement of technology resources and AI integration (Knox, 2020; OECD, 2022). Therefore, education policy is conceptualized in this study as a contextual factor that exerts influences on technology adoption.

Furthermore, UTAUT has limited consideration of prior experiences and technology adoption requires sustained use. In educational contexts, particularly for pre-service teachers, early exposure to digital tools and repeated practice during training can gradually transform intentional use into habitual use, thereby shaping their future teaching practices (Bervell et al., 2022; Gardner et al., 2022). Considering the growing presence of AI tools in teacher education programs, AI usage habit is introduced in this study to capture the relationship between prior-AI-related experiences and music student teachers’ intentions to integrate AI into future classrooms.

By incorporating both education policy and AI usage habit into the UTAUT framework, the extended model will explore the relationships between institutional structures, habitual use, and individual perceptions in shaping music student teachers’ intentions to use AI. Considering the increasing AI integration into teaching, this study will make contribution to the design of teacher training programs and provide guidance for governments and educational institutions to prepare future educators for AI-enhanced learning environments (Wang Y. et al., 2025).

#### Performance expectancy (PE)

Performance expectancy is originally defined as the extent to which individuals believe that applying a particular technology will improve their performance at work (Venkatesh et al., 2003). It refers to music student teachers’ beliefs about the usefulness and effectiveness of AI in improving their teaching performance in the present study (Venkatesh et al., 2012; Wong et al., 2013). Previous studies indicated a positive association between perceived usefulness and willingness across various user groups (Ogegbo et al., 2024; Dwivedi et al., 2019). However, research in educational contexts has yielded various findings. For instance, a plethora of existing studies reported no significant relationship between perceived usefulness and students’ willingness to use Google classroom or other e-learning systems (Alotumi, 2022; Bervell et al., 2022; Hunde et al., 2023). In contrast, studies focusing on Chinese teachers found that performance expectancy is an important determinant of integrating technologies into teaching (Wijaya et al., 2022; Huang et al., 2025). Based on this evidence, we proposed the following hypothesis:

*H1*: Performance expectancy (PE) significantly influences music student teachers’ behavioral intention (BI) to apply AI tools in classrooms.

#### Effort expectancy (EE)

The degree of perceived ease related to technology use is defined as effort expectancy (Venkatesh et al., 2003). It reflects pre-service music teachers’ perceived ease of integrating AI tools into music teaching in the current study. Prior studies have recognized effort expectancy as an important predictor of teachers’ willingness of using educational technologies (Wijaya et al., 2022; Kim and Lee, 2022). For example, Shah et al. (2021) and Wong et al. (2013) reported that perceived ease of use played an important role in shaping teachers’ use of technologies (e.g., ICT, interactive whiteboard). Conversely, some studies found non-significant relationships between teachers’ intention and their effort expectancy of integrating technology into classrooms (Liu et al., 2025; Tseng et al., 2022). Despite these inconsistencies, research explored the correlation of effort expectancy and behavioral intention among music student teachers remains limited. Therefore, based on the existing literature, we posited:

*H2*: Effort expectancy (EE) significantly affects music student teachers’ behavioral willingness of using AI in education.

#### Social influence (SI)

Social influence is usually defined as how individuals perceive important others believe they should apply certain technology (Venkatesh et al., 2003). Prior research conceptualizes social influence as a kind of pressure on individuals to engage or not engage in specific behaviors (Shah et al., 2021). In the current study, it refers to the extent to which music student teachers perceive expectations and support from important others regarding AI use in teaching. Previous studies demonstrated the critical role of important others in shaping student teachers’ perceptions toward technology integration (Grassini, 2023). Similarly, Kim et al. (2020) found that encouragement and feedback from important others (e.g., mentors and peers) who have successfully integrated AI can substantially affect teachers’ decisions on AI. Correspondingly, we proposed that:

*H3*: Social influence (SI) significantly influences music student teachers’ behavioral intention (BI) of AI use in classrooms.

#### Facilitating conditions (FC)

Facilitating conditions is commonly defined as the degree to which individuals think that organizations support their implementation of technology (Adigun et al., 2025). In the present study, it concerns music student teachers’ perceptions of whether their environments provide adequate support for AI in their practices. Supportive conditions in education contexts (e.g., teacher training, technical assistance and training, access to resources) are recognized as critical factors for AI use (Bearman et al., 2023). It is in accordance with prior findings that student teachers tend to integrate AI tools into future teaching when they receive necessary and sufficient institutional support for AI use (Zhai et al., 2021). Riady et al. (2022), for instance, investigated 1,249 Indonesian teachers and reported the significant relationship between facilitating conditions and teachers’ intentions of using technology. However, some previous studies demonstrated inconsistent findings. Tseng et al. (2022) found a significantly direct effect of facilitating conditions on teachers’ willingness of adopting educational technologies, whereas Alotumi (2022) observed no significant predictive effect. Given these mixed findings, the present study proposed the following hypothesis:

*H4*: Facilitating conditions (FC) significantly influences music student teachers’ behavioral intentions (BI) of using AI in music education.

#### Education policy (EP)

Educational policy represents an important component of national agendas around the world as it addresses issues including politics, education, technology, and culture (Kim and Lee, 2022). In the present study it refers to music student teachers’ perceived policy influence related to AI use in the classrooms. In China, the government regularly issues policies and guidelines that focus on the integration of emerging technologies into teaching in response to societal, educational, and cultural needs (Xue et al., 2021). Prior research suggests that important agents (e.g., policymakers) may affect teachers’ perceptions and behaviors of technology use (Huang et al., 2019). Li (2018) reported that based on the top-level design of the education policy in China, social and institutional forces provide various support (e.g., software resources, professional training, and online platforms) to foster technology-enhanced teaching. Such support is expected to improve facilitating conditions and increase teachers’ effort expectancy toward AI tools (Li et al., 2022; Sang et al., 2023). Moreover, prior literature indicated that education policy can shape institutional norms, peer expectations (OECD, 2022) and promote student teachers’ habitual use of AI tools (Gardner et al., 2022). Accordingly, we posited the following hypotheses:

*H5a*: Education policy (EP) significantly influences music student teachers behavioral willingness of using AI tools in music classes (BI).

*H5b*: Education policy (EP) positively influences facilitating conditions (FC), effort expectancy (EE), social influences (SI) and AI usage habit (AUH), which in turn significantly influence music student teachers’ behavioral intentions of AI use in music classrooms (BI).

#### AI usage habit (AUH)

Habit is conceptualized as the extent of preference of performing particular behaviors automatically (Venkatesh et al., 2012). Some information system studies conceptualized habit from two perspectives: individuals’ previous behaviors and automaticity (Kim and Lee, 2022). The present study adopts both perspectives and the pre-service music teachers’ AI usage habit is defined as the degree to which they habitually use AI tools in music education as a result of their previous usage experiences. Existing literature highlights that the habit of technology use is positively associated with individuals’ willingness and actual behaviors (Bervell et al., 2022). Accordingly, we posited:

*H6*: AI usage habit (AUH) is positively associated with music student teachers’ integration of AI tools into music classrooms.

#### Behavior intention to use AI (BI)

In UTAUT model, behavioral intention refers to individuals’ willingness of adopting and using a particular technology in the future (Venkatesh et al., 2003). In the current study, it refers to music student teachers’ willingness of integrating educational AI tools into teaching. Behavioral intention is commonly recognized as the strongest predictor of individuals’ actual technology use and has been reported to significantly affect actual behaviors (Kim and Lee, 2022). In the original UTAUT model, behavioral intention is influenced by core determinants while facilitating conditions directly influence actual use (Venkatesh et al., 2012). The proposed model in the current study will examine how additional factors (e.g., educational policy and AI usage habit) interact with UTAUT constructs to influence Chinese music student teachers’ willingness of AI use in their teaching practices.

## Method

### Sample and data collection

**Figure 1:**
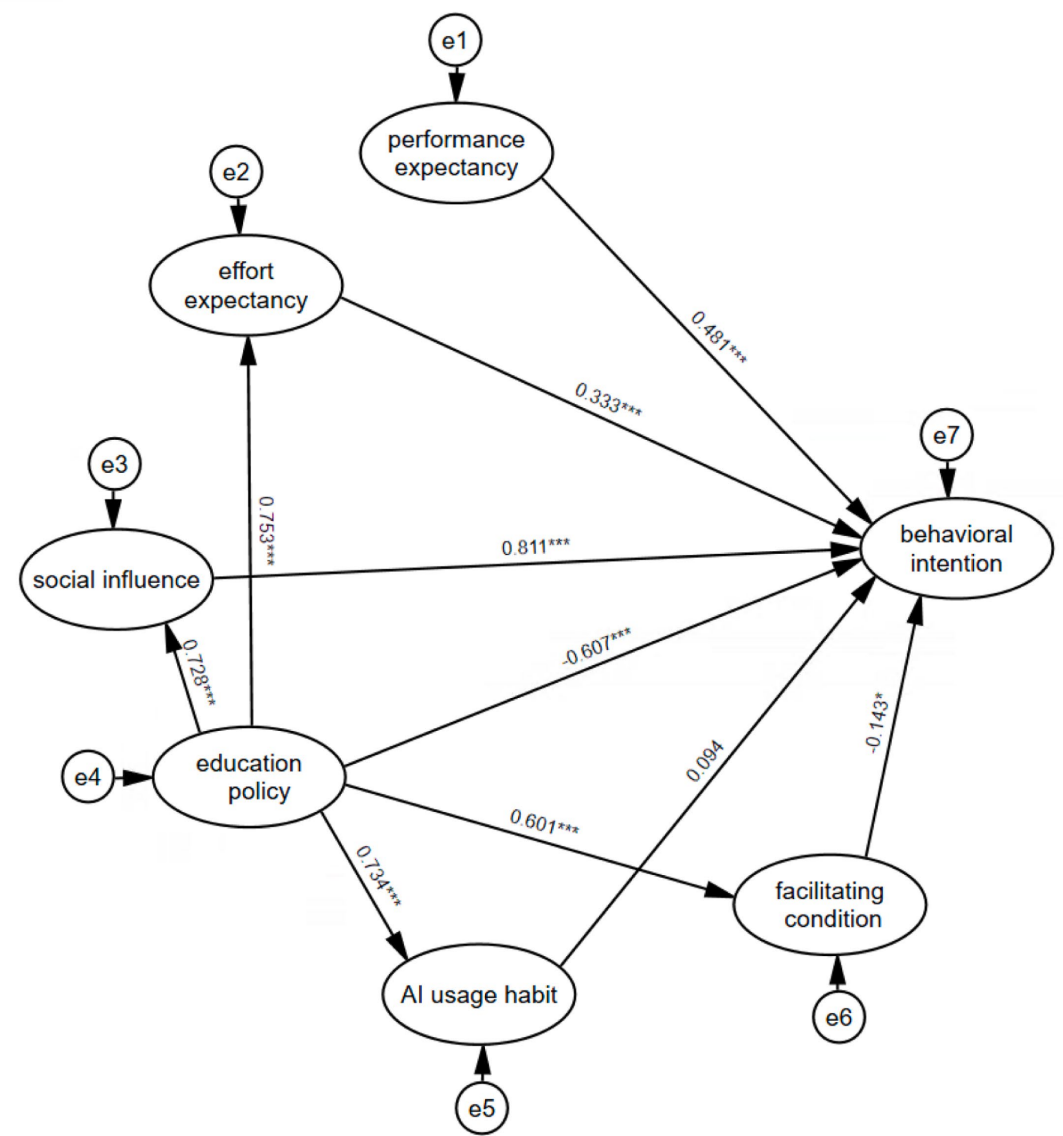
Path analysis of the research model. ***p < 0.001, *p < 0.05.

An online survey was administered to obtain the information used to examine the proposed model in this study (see Figure 1). The investigation received ethical approval from the Ethics Committee of Zhengzhou University of Science and Technology. The research target was music student teachers. The survey was distributed and data was collected in September 2025 and included 393 participants. From the outset, we informed all the participants that they participated in this study voluntarily and they could withdraw from the investigation at any point. Music student teachers who agreed to participate were sent survey package online, and completed questionnaires were collected after 1 week of the delivery. Except for 23 incomplete and invalid respondents, 370 valid cases were retained for following formal data analysis. Among 370 participants, 327 (88.4%) were female and 43 (11.6%) were male. This gender distribution is consistent with the female-dominant enrollment trend in teacher education (Wong et al., 2013). With respect to educational background, 212 (57.3%) were diploma’s students, while 158 (42.7%) were Bachelor’s students. Regarding previous AI learning experiences, 71.9% of respondents had related AI learning experiences, and 28.1% never had. 112 had prior school-based placement. 107 frequently use AI tools to facilitate their lesson preparation and study. Participants spent approximately 10–20 min to complete the questionnaire. Participants’ demographic information is presented in Table 1.

**Table 1:** Participants by demographic characteristics (*N* = 370).

Background variables	Categories	Frequency	Percentage
Gender	Female	327	88.4
	Male	43	11.6
Certificate	Diploma	212	57.3
	Bachelor	158	42.7
Placement	Yes	266	71.9
	No	104	28.1
Previous AI learning experience	Yes	112	30.3
	No	258	69.7
Frequency	never	12	3.2
	seldom	128	34.6
	sometimes	123	33.2
	often	98	26.5
	always	9	2.4

### Measurement

The online questionnaire consisted of two sections. Participants demographics were gathered in the first section. The second section was about seven core constructs of the proposed model and it originally included 27 items, namely performance expectancy (3 items), AI usage habit (4 items), effort expectancy (5 items), education policy (4 items), social influence (4 items), facilitating conditions (4 items), and behavioral intention (3 items). Those question items are adapted from previous research (Venkatesh et al., 2012; Kim and Lee, 2022) and revised to fit the context of music student teachers’ AI use in educational practices. All items used a five-point Likert scale, ranging from 1 (strongly disagree) to 5 (strongly agree). Before the main data collection, two experts in music education and AI in education reviewed the questionnaire to evaluate the content of the questionnaire and then a pilot test was subsequently conducted to assess the quality of the questionnaire. Five items were removed because of insufficient reliability and low factor loadings.

### Data analysis

The collected information was analyzed by SPSS 26 and AMOS 30 and structural equation modeling (SEM) procedures were used. SEM is recognized for its capacity to evaluate relationships among the determinants of individuals’ willingness of integrating technology in educational contexts (Wang and Shih, 2009; Wong et al., 2013). In the current study, SEM was employed to examine the proposed UTAUT model and to explore whether the path coefficients are significant.

## Results

Table 2 presented the descriptive statistics of the constructs. All the seven constructs have mean scores above the midpoint of 3, ranging from 3.39 (EP) to 4.06 (PE). Skewness and Kurtosis coefficients all fell within acceptable ranges and this indicates a multivariate normality (Cohen et al., 2003).

**Table 2:** Descriptive statistics.

Construct	No. of items	Mean	SD	Skewness	Kurtosis
PE	3	4.06	0.62	0.01	−0.65
EE	3	3.60	0.76	−0.34	−0.23
SI	3	3.79	0.62	0.29	−0.58
EP	4	3.39	0.98	−0.31	−0.73
AUH	3	3.57	0.84	−0.22	−0.00
FC	3	3.62	1.02	−0.94	0.82
BI	3	3.94	0.63	0.08	−0.81

PE = Performance expectancy, EE = Effort expectancy, SI = Social influence, EP = Education policy, AUH = AI usage habit, FC = Facilitating conditions, BI = Behavioral intention.

### Evaluation of the measurement model

Tables 3, 4 summarized the measurement results. To examine the adequacy of the extended UTAUT model, the evaluation should satisfy the following criteria: composite reliability (CR) and Cronbach’s *α* values above 0.7 (Hair et al., 2009; Martínez-López et al., 2013), standardized factor loadings above 0.5, and average variance extracted (AVE) values above 0.5 (Fornell and Larcker, 1981). As shown in Table 3, all the factor loadings were from 0.616 (SI3) to 0.901 (FC1), higher than 0.5, and this confirmed acceptable indicator reliability. Cronbach’s α of each construct ranged from 0.730 (social influence) to 0.918 (facilitating conditions) and exceeded the recommended benchmark (0.70), demonstrating great composite reliability of each construct (Hair et al., 2009). Discriminant validity was presented in Table 4 and supported by the criterion as the square root of the AVE for each construct was greater than its correlations with other constructs (Fornell and Larcker, 1981). The AVE for each construct was above 0.5 except that social influence (0.479) and AI usage habit (0.499) were slightly below the recommended threshold. Some research indicated that AVE values between 0.4 and 0.5 are acceptable when composite reliability exceeds 0.6 (Fornell and Larcker, 1981; Durgapal and Saraswat, 2019; Verhoef et al., 2002). Therefore, all the recommended criteria are fulfilled and the measurement model meets the recommended validity criteria.

**Table 3:** Construct reliability and convergent validity.

Construct/indicator	QI	FL	CR	AVE	α
Performance expectancy	PE1	0.692	0.764	0.519	0.792
	PE2	0.763			
	PE3	0.704			
Effort expectancy	EE1	0.815	0.825	0.611	0.825
	EE2	0.748			
	EE3	0.781			
Social influence	SI1	0.682	0.732	**0.479**	0.730
	SI2	0.616			
	SI3	0.770			
Education policy	EP1	0.794	0.854	0.595	0.878
	EP2	0.823			
	EP3	0.808			
	EP4	0.648			
AI usage habit	AUH1	0.691	0.747	**0.499**	0.746
	AUH2	0.610			
	AUH3	0.804			
Facilitating conditions	FC1	0.901	0.919	0.790	0.918
	FC2	0.884			
	FC3	0.881			
Behavioral intention	BI1	0.703	0.799	0.571	0.816
	BI2	0.768			
	BI3	0.793			

QI = questionnaire items, FL = factor loading, CR = composite reliability, AVE = average variance extracted, α = Cronbach’s alpha.

**Table 4:** Correlation matrics and discriminant validity.

Construct	PE	EE	SI	EP	AUH	FC	BI
PE	**0.903**						
EE	0.463	**0.880**					
SI	0.575	0.662	**0.826**				
EP	0.242	0.666	0.601	**0.849**			
AUH	0.336	0.626	0.683	0.792	**0.675**		
FC	0.343	0.758	0.663	0.539	0.658	**0.949**	
BI	0.699	0.479	0.727	0.346	0.459	0.427	**0.914**

Fornell-Larcker criterion for discriminant validity.

### Evaluation of structural model

The study performed a path analysis to examine the overall goodness-of fit of the proposed UTAUT model. AMOS 30.0 was performed and maximum likelihood estimation (MLE) procedure was used for model estimation. Multiple model-fit indices, x^2^/df, TLI, CFI, GFI, NFI, IFI and RMSEA, were used. As shown in Table 5, the proposed model demonstrated an acceptable to good overall fit. x^2^/df was 2.650. All the fit statistics except GFI and NFI, exceeded 0.9. RMSEA is valued at 0.067, lower than 0.08. Based on commonly recommended cut-off values (Schumacker and Lomax, 2010; Hair et al., 2009), the GFI (0.879) and NFI (0.876) values remain within the acceptable range (≧0.85), indicating that the overall model fit is adequate.

**Table 5:** Fit indices for the path model.

Model fit index	Values	Recommended guidelines
x^2^/df (deg. of freedom)	2.650	<3
TLI	0.905	≧0.9
CFI	0.918	≧0.9
GFI	**0.879**	≧0.9
NFI	**0.876**	≧0.9
IFI	0.919	≧0.9
RMSEA	0.067	<0.08

### Hypotheses testing

The standardized path coefficients for the extended UTAUT model are presented in Figure 1. As shown in the figure, performance expectancy (*β* = 0.481, *p* < 0.001), effort expectancy (*β* = 0.333, *p* < 0.001), social influence (*β* = 0.811, *p* < 0.001), facilitating conditions (*β* = −0.143, *p* < 0.05), and education policy (*β* = −0.607, *p* < 0.001) all exerted statistically significant influences. Therefore, hypotheses H1–H5 were supported (Table 6). However, AI usage habit (*β* = 0.094, *p* > 0.10) had no significant influence on music student teachers’ intentions and H6 was not supported.

**Table 6:** Summary of hypotheses tests.

Hypotheses	Path	Path coefficient	*p*-value	Results
H1	PE → BI	0.481	***	Supported
H2	EE → BI	0.333	***	Supported
H3	SI → BI	0.811	***	Supported
H4	FC → BI	−0.143	0.029*	Supported
H5	EP → BI	−0.607	***	Supported
H6	AUH → BI	0.094	0.320	Not supported

Standardized estimate are shown. ***p < 0.001, *p < 0.05.

To evaluate explanatory capability of the extended UTAUT model, the R^2^ value for pre-service music teachers’ intentions was examined. The R^2^ value is widely applied to indicate the extent to which variables can explain (Kim and Lee, 2022). The five significant predictors jointly accounted for 62.4% of its variance (*R*^2^ = 0.624). According to Chin’s (1998) criteria, the obtained R^2^ approaches the threshold for substantial predictive power (*R*^2^ > 0.67). This indicates that the proposed model offers strong predictive relevance.

To clarify the role of education policy, Table 7 demonstrates its direct, indirect and total effects on intentions. Except for the significant negative direct impact (*β* = −0.607, *p* < 0.001), education policy produced a strong indirect effect (*β* = 0.824, *p* < 0.001) and total effect on intentions (*β* = 0.216, *p* < 0.001). As detailed in Table 8, education policy produced significant positive indirect effects through effort expectancy (*β* = 0.248, *p* < 0.05) and through social influence (*β* = 0.591, *p* < 0.001). The overall association between education policy and intention becomes positive. This indicates the complex role that education policy plays in shaping music student teachers’ willingness of using AI in music classrooms.

**Table 7:** Direct, indirect and total effects - estimates.

Predictors	Criterion variable		
	BI		
	Direct effects	Indirect effects	Total effects
EP	−0.607***	0.824***	0.216***

Standardized estimates are shown, ***p < 0.001.

**Table 8:** Summary of hypotheses tests for indirect effects of EP.

Hypotheses	Path	*β*	p-value two tailed	BC 95% CI		Results
				Lower	Upper	
H5b	EP → EE → BI	0.248	0.005**	0.043	0.270	Supported
	EP → SI → BI	0.591	0.000***	0.210	0.613	Supported
	EP → FC → BI	−0.084	0.064	−0.105	0.003	Not supported
	EP → AUH → BI	0.066	0.303	−0.038	0.135	Not supported

BC = bias corrected. 5,000 bootstrap samples, ***p < 0.001, **p < 0.01.

## Discussion

### Direct effects of constructs on AI use intentions

The present study investigated the factors influencing music student teachers’ willingness of adopting AI in music classrooms based on an extended UTAUT model. The proposed model explained 62.4% of the variance in music student teachers’ willingness and there are five variables, performance expectancy, effort expectancy, social influence, facilitating conditions, and education policy, emerging as significant determinants. The measurement and structural model results also confirm and validate the proposed model. These findings yielded several implications for music teacher education and AI-supported teaching.

Performance expectancy, effort expectancy and social influence exerted significant and positive influences on music student teachers’ willingness of using AI and this aligned with existing literature (Wong et al., 2013; Kim and Lee, 2022; Tseng et al., 2022). Performance expectancy was the second most influential predictor, following social influence. This suggested that music student teachers tend to adopt AI technology when they see advantages and benefits and link such values to AI utilization. Unlike other subjects, music teaching relies heavily on specialized tasks such as auditory discrimination and musical performance (Han, 2025). From the results, it is clear that music student teachers appear motivated to use AI tools when they believe AI can help them enhance these tasks. Therefore, policymakers and curriculum designers should not only put emphasis on the usefulness of AI in practices and demonstrate it to student teachers, but also design AI training that tailored to the artistic and technical demands of music teaching.

The significant influence of effort expectancy suggests that music student teachers tend to implement AI in future music classrooms when they feel that AI tools are easy for them to apply, which is in accordance with early studies (Adigun et al., 2025; Hu et al., 2025). Therefore, it is important to develop pre-service music teachers’ positive attitudes of ease of use of educational AI tools at the early stage of implementation (Wong et al., 2013). As AI music tools often involve complex interfaces such as AI scoring systems and performance recognition algorithms (Hosseini et al., 2024), teacher education providers should reduce the cognitive load of learning and increase hands-on demonstrations and practices with music-focused technologies.

Social influence is the strongest predictor of music student teachers’ willingness of using AI in classrooms, in alignment with prior UTAUT research within teacher education contexts (Saeed Al-Maroof et al., 2020). This indicates that music student teachers’ decisions about AI in future practices are highly influenced by their mentors, peers and institutional expectations (Hu et al., 2025; Islamoglu et al., 2021). Moreover, the collectivistic cultural patterns in China amplify the impact of social expectation from mentors, supervisors and institutions which can exert additional pressure on student teachers (Zhao et al., 2021). Therefore, to develop the innovation in music education, policymakers should also focus on teacher education practitioners and with their endorsement, music student teachers tend to integrate AI into music learning processes.

The result of facilitating conditions is consistent with previous UTAUT research (Wang Y. et al., 2025) and shows that music student teachers’ willingness is closely related to institutions’ support and commitment toward the AI applications (Kim and Lee, 2022). However, the finding suggests that facilitating conditions exerted significant negative impact. This aligns with psychological reactance theory which suggests that external imposed requirements or expectations can reduce individuals’ willingness even when resources are provided (Rosenberg and Siegel, 2018). For novice or student teachers, institutional provision of AI resources or mandated training can be interpreted as external pressure rather than support (Guo, 2024). Although Chinese government makes a series of measures to establish infrastructures and curricula capable of adapting AI in education systems (Knox, 2020), such measures may not enhance teachers’ willingness of implementing AI into practices. Therefore, policymakers and teacher education practitioners need to frame AI as optional rather than mandatory enhancement, especially during the early process of adoption. In the meanwhile, teacher education programs can provide AI-related learning opportunities as effective components to foster student teachers’ exploration of AI applications and accept their choices of using AI or not to reduce psychological reactance and develop more attitude-driven adoption.

This study revised UTAUT model by adding education policy and habit of using AI to suit an educational context in China. Contrary to expectations, AI usage habit did not have a significant effect on music student teachers’ behavioral intentions to implement AI in practices while education policy exerted significant negative direct effect on student teachers’ behavioral willingness. This is consistent with the fact that AI tools in teaching are still emerging and pre-service music teachers usually have limited experiences integrating AI into authentic classroom settings (Sun et al., 2024). Student teachers’ implementation of AI tools tends to be assignment-driven, or influenced by mentors rather than habitual. Habit typically becomes a strong predictor when individuals have extensive past experience and stable usage (Alalwan et al., 2015). Compared with in-service teachers, student teachers usually lack repeated practice in realistic settings to reshape and update their existing habit (Hobbiss et al., 2021). Therefore, longitudinal research could be used to explore whether habit becomes more influential once AI music educational tools are widely implemented across schools and student teachers have more exposure to actual AI-enhanced teaching settings.

### A dual-path mechanism of education policy

Education policy was found to directly and negatively influence music student teachers’ intention to use AI, consistent with previous studies. Kim and Park (2023) found that policies may trigger individuals’ emotional resistance and innovation resistance and negatively influence their behavioral intention. Wang J. et al. (2025) demonstrated that music teachers may reduce their willingness of technology adoption and resist the change of using technology when they are required to do so. This suggests that top-down policy may create additional workload and pressure and may directly suppress teachers’ intention to adopt AI into teaching (Lu and Wang, 2023).

In addition, this study reveals that education policy also exerts positive but indirect influence on student teachers’ willingness of using AI through effort expectancy and social influence. Although policy measures may create pressures and impact teachers’ intentions, they can simultaneously improve institutional resources and professional development through infrastructure and training (Sang et al., 2023; Watkins, 2022). This can increase student teachers’ perceptions of ease of use and then strengthen effort expectancy (Feng et al., 2025). Furthermore, policy initiatives can shape social norms and expectations with teacher education programs, which can amplify social influence and indirectly promote teachers’ technology use (Shahzad et al., 2024).

These findings highlight the dual and layered influence of policy influence in teacher education. At the direct level, policy-related expectations may be perceived by student teachers as external pressure and potentially elicit their psychological resistance. At the indirect level, education policy functions as structural support that shapes normative expectations and allocates resources and training. Therefore, the effects of education policy on technology acceptance are not unidirectional but operate through multiple interacting pathways. This not only clarifies why education policy can simultaneously constrain and promote AI adoption but also highlights the importance of considering both individual perceptions and institutional mechanisms when examining AI integration in teacher education.

## Conclusion

This study explored determinants that influence music student teachers’ intentions of integrating AI into practices through an extended UTAUT framework. By adding education policy and AI usage habit into the theoretical model, the findings of this study offer a more comprehensive understanding of how future educators perceive and adopt emerging technologies. Another important contribution of this research is to reveal the dual-path role of education policy and this underscores the importance of considering both individuals’ perceptions and institutional mechanisms when policymakers design AI-related educational reforms. The findings also emphasize the need for teacher education practitioners to provide discipline-specific AI training. Furthermore, this study deepens the understanding of music student teachers’ AI acceptance and offers a perspective for future research to examine how AI can shape teacher education within unique contexts.

The present study has several limitations. First, self-reported information is collected through an online questionnaire which may lead to social desirability bias or inaccurate self-assessment. Although structural equation modeling allows for testing theoretically grounded directional relationships, longitudinal or mixed-methods research could be used to investigate how student teachers’ perceptions and actual AI use change over time. Second, education policy referred to perceived policy influence rather than objective exposure to specific policy or requirements. Thus, this construct reflects individual interpretations which may vary across institutions. Future studies could integrate policy document analysis to better connect macro-level policy with individual-level responses. Additionally, the sample was drawn from pre-service music teachers in China and this may restrict the generalizability of the findings to other areas, disciplines or cultural contexts. Comparative studies across regions, subjects or cultural backgrounds can examine how contextual differences shape AI adoption among student teachers and test the cross-cultural applicability of the extended UTAUT model. Finally, as habit formation and technology acceptance are likely to develop with increased teaching experience, future research could extend this model to in-service teachers and examine potential moderating effects of individual characteristics (e.g., prior AI experience and AI competence).

## Data Availability

The original contributions presented in the study are included in the article/supplementary material, further inquiries can be directed to the corresponding author.
